# Applications of DESI and DART Mass Spectrometry in Forensic Science

**DOI:** 10.1021/jasms.5c00175

**Published:** 2025-10-01

**Authors:** Nathália dos S. Conceição, Alan R. Pereira, Daniel S. Trancoso, João Victor M. de Almeida, Nicole Souza do Carmo, Nayara A. dos Santos, Hildegardo S. França, Marc Yves Chalom, Wanderson Romão

**Affiliations:** † Federal University of Espírito Santo, Fernando Ferrari Avenue, 514, Goiabeiras, Vitória-ES 29075-910, Brazil; ‡ Federal Institute of Espírito Santo, Ministro Salgado Filho Avenue, Soteco, Vila Velha-ES 29106-010, Brazil; § 200997 Bruker Corporation, Bosque da Saúde Lane, São Paulo-SP, 04142-082, Brazil

## Abstract

Ambient Mass Spectrometry (AMS) has revolutionized forensic analysis by enabling rapid and direct detection of chemical compounds on complex surfaces with minimal or no sample preparation. Among AMS techniques, desorption electrospray ionization (DESI) and direct analysis in real time (DART) mass spectrometry have emerged as powerful analytical tools due to their speed, sensitivity, and versatility. This review examines the major applications of DESI and DART in forensic science, including the detection of explosives, gunshot residue, illicit drugs, inks, biological fluids, and latent fingerprints. The principles of each technique are briefly discussed, followed by a comparative analysis of their advantages, limitations, and performances in various forensic scenarios. Recent developments, practical considerations for implementation, and future perspectives are also addressed, highlighting the growing impact of DESI and DART in real-time forensic investigations and evidence processing.

## Graphical abstract

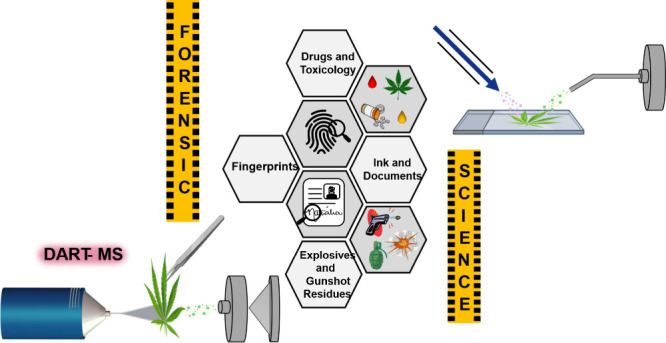

## 1 Introduction

Mass spectrometry (MS) is one of the most powerful analytical techniques in forensic science, enabling the accurate identification, quantification, and characterization of a wide range of compounds in criminal evidence. Its application is particularly relevant in the analysis of controlled substances, explosives, gunshot residue, biological traces and forged documents, making it an essential tool in investigations and trials.^1^

For the analysis to take place, the analyte must be converted to ions in the gas phase, a process carried out by different ionization methods. However, conventional techniques often require extensive sample preparation, such as extraction and chromatographic separation, which can be time-consuming and impractical in emergency forensic analysis.^2,3^ In addition, the ionization process plays a key role in mass spectrometry, directly influencing the efficiency of the analysis and the quality of the results obtained.^4^

Advances in ionization techniques have revolutionized mass spectrometry by enabling faster, more efficient, and less labor-intensive analyses. Among the most significant developments is AMS, which permits the direct ionization and detection of samples in their native state, eliminating the need for complex sample preparation. This innovation has provided substantial benefits to forensic science, facilitating the rapid detection of suspected substances at crime scenes, airports, in toxicological investigations, and across a range of forensic context.^5^ The growing relevance of AMS in this field is evidenced by the increasing number of publications on the subject. As illustrated in Figure 1, more than 4000 studies involving the analysis of forensic matrices using AMS have been published over the past decade, with over 50% of these studies focusing on the detection of illicit drugs.

**Figure 1:**
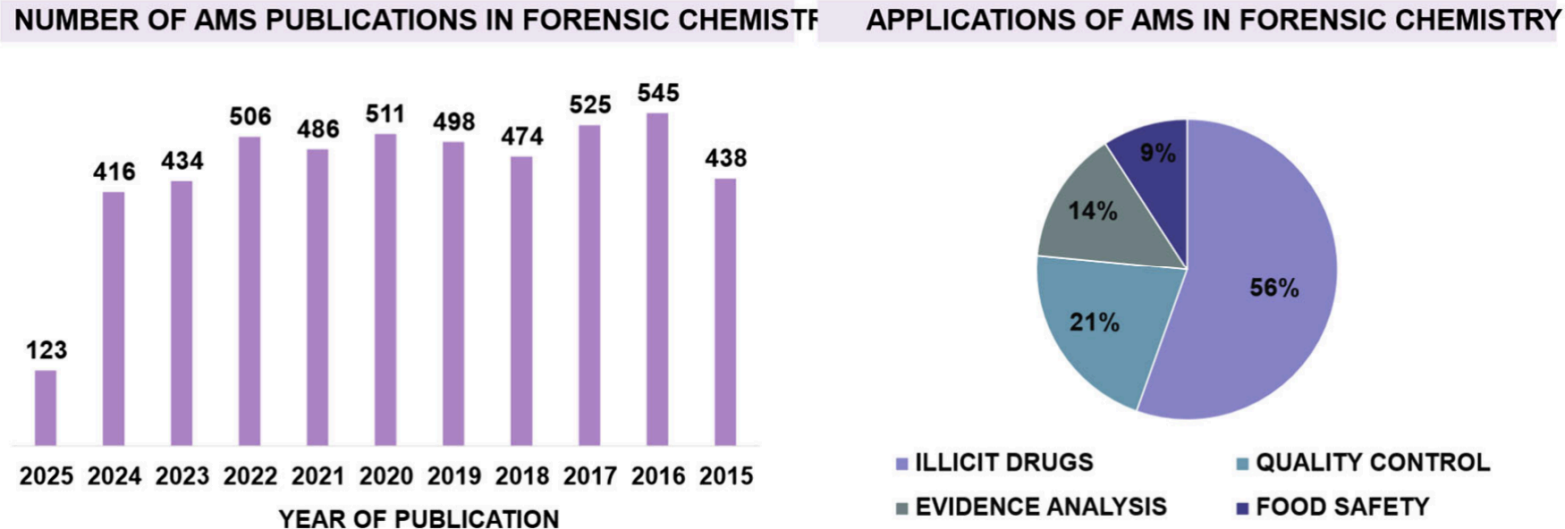
Representative graph showing the number of publications over the past decade involving the use of AMS in Forensic Chemistry (search terms: “Ambient Mass Spectrometry” and “Forensic Chemistry”), as well as the main application areas of AMS within the field. Source: Web of Science, accessed on April 25, 2025.

AMS was first mentioned in 1998 when Fenn anticipated the concept of Paper Spray Mass Spectrometry (PS-MS) in his patent by describing a direct ionization method using cellulose-based materials.^6^ However, the first documented study on the application of PS-MS was published in 2010 by Liu et al. Furthermore, the DART source was first developed in 2003 and became the first commercially released AMS system in 2005.^7,8^ However, the DESI technique, developed by Takáts et al. in 2004, was the first ambient ionization method to be formally reported in the scientific literature.^9,10^

AMS can be classified into two main groups: I - Techniques based on Electrospray Ionization (ESI), such as Desorption Electrospray Ionization (DESI),^10^ Extractive Electrospray Ionization (EESI),^11^ Paper Spray Ionization (PSI),^9^ Probe Electrospray Ionization (PESI),^12^ Easy Ambient Sonic-Spray Ionization (EASI),^13^ and Laser Ablation Electrospray Ionization (LAESI),^14^ and II - Techniques based on Atmospheric Pressure Chemical Ionization (APCI), including Direct Analysis in Real Time (DART),^15^ Low Temperature Plasma Ionization (LTP),^16^ Dielectric Barrier Discharge Ionization (DBDI),^17^ Desorption Atmospheric Pressure Chemical Ionization (DAPCI),^18^ and Atmospheric Solids Analysis Probe (ASAP).^19^ Figure 2 presents the original publication for each of the aforementioned techniques, as well as their year of development and the responsible author.

**Figure 2:**
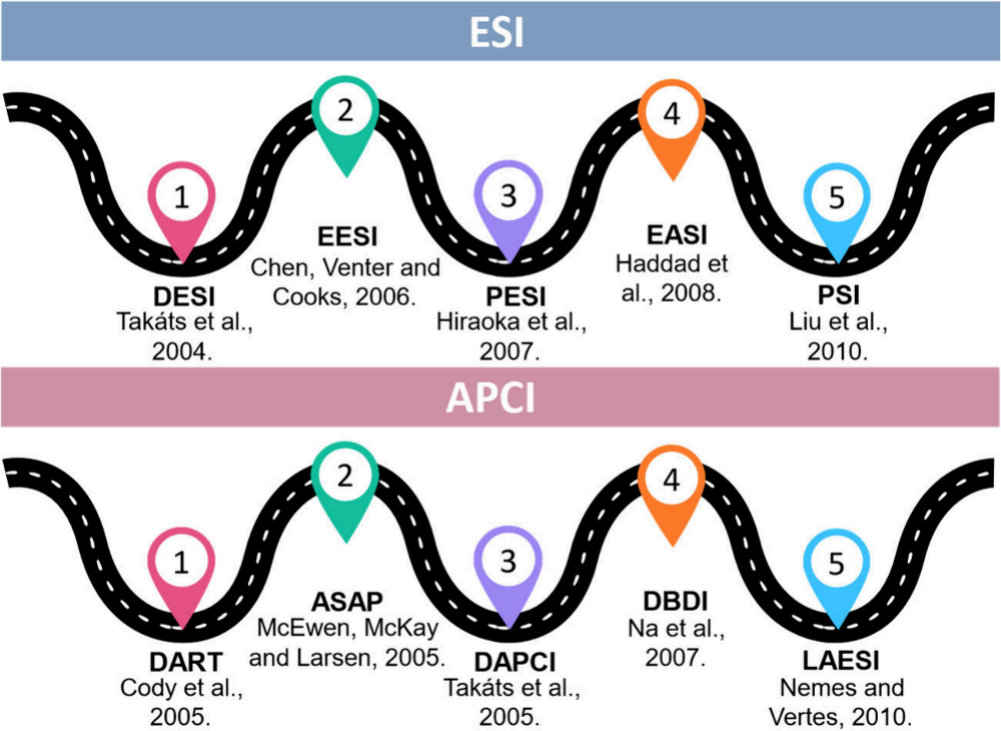
Ambient mass spectrometry techniques based on electrospray ionization and atmospheric pressure chemical ionization consider the respective publication of origin.

AMS techniques include DESI, introduced by Cooks and collaborators in 2004, and DART, developed by Cody and collaborators in 2005. Both techniques were designed to allow fast and reliable analysis, reducing sample processing time and ensuring greater reproducibility of the results. In recent years, these techniques have been consolidated as essential tools in forensic science.^15,20,21^

In order to check how these two techniques are being used in terms of the number of publications, a search was carried out in the Web of Science database using the “title”, “abstract” and “author keywords” filters for the periods 2015–2025 (term: “DESI”) and 2015–2024 (term: “DART”). The results of this search are seen in Figure 3. During the period analyzed, 928 articles applying the DESI technique and 1076 articles on DART were found.

**Figure 3:**
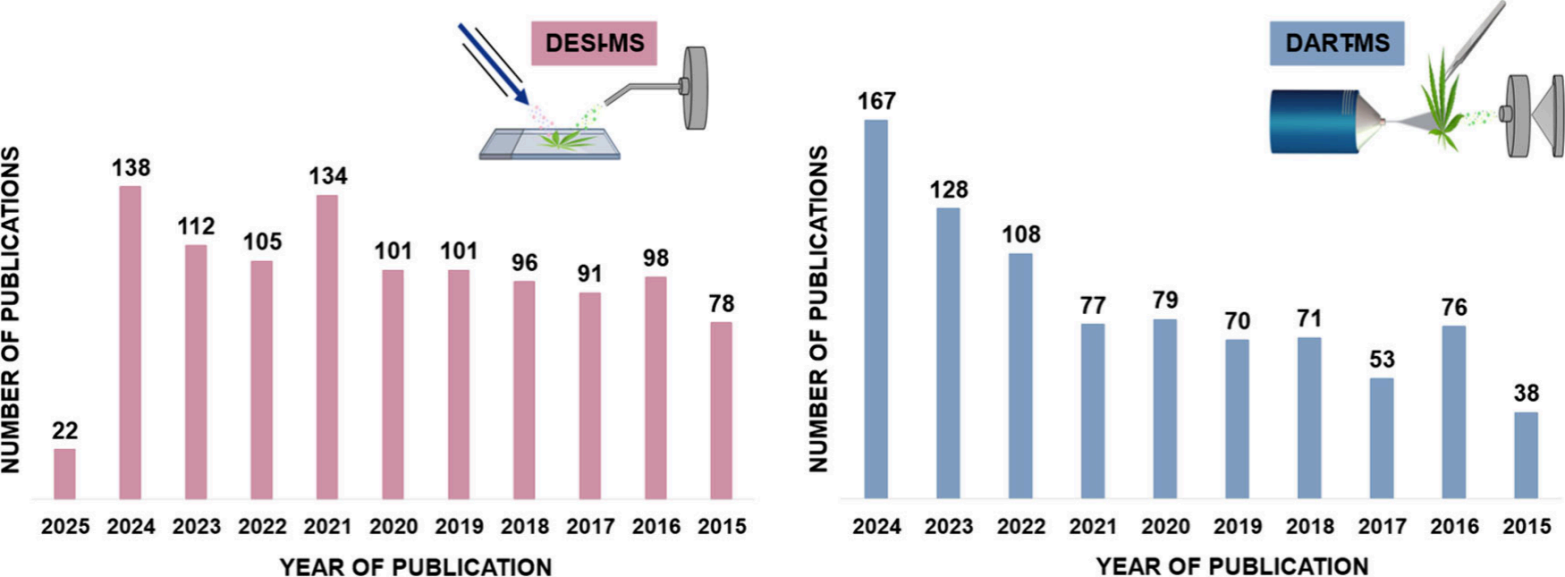
Representative graph showing the number of papers published using the DESI (2015–2025) and DART (2015–2025) techniques. Source: Web of Science, accessed on March 19, 2025.

Bibliometric data also show the growing relevance of these techniques, based on the number of publications and the citation of their foundational articles. Figure 4 shows the 10 most cited authors for each of the techniques, highlighting the original articles by Cooks et al. (2004) on DESI and Cody et al. (2005) on DART, which are the most cited among the published studies. These results indicate not only the growing interest in MSA, but also the significant impact of its pioneers, Cooks and Cody, on the advancement of these analytical approaches.

**Figure 4:**
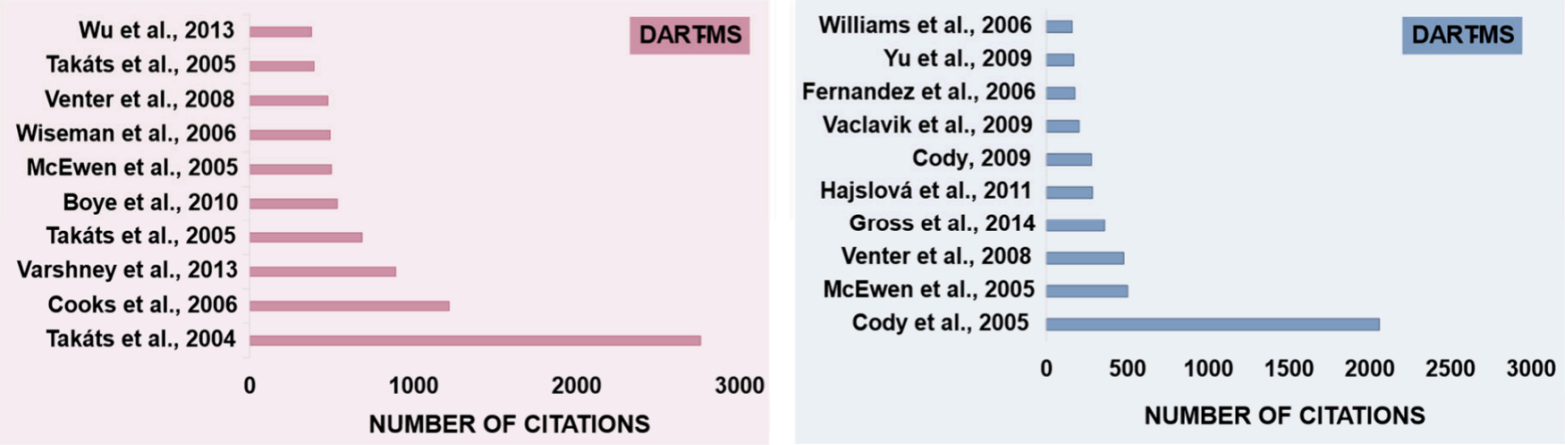
Representative graph showing the ten most cited articles and their respective number of citations, referring to the DESI-MS and DART-MS techniques. Source: Web of Science, accessed on March 19, 2025.

In short, the bibliometric data show the growing importance of AMS in forensic science, highlighting the significant impact of DESI and DART techniques. The increase in the number of citations and publications demonstrates the success of these methodologies in detecting illicit substances, explosives, and biological residues. The ability to analyze samples with minimal preparation and provide results in real time reinforces their relevance in criminal investigations. Since its introduction, AMS has constantly evolved, with the emergence of new variations based on DESI and DART, consolidating itself as a modern, versatile, and efficient approach in the context of mass spectrometry applied to forensics.

## 2 Principles of DESI-MS and DART-MS Techniques

### 2.1 Desorption Electrospray Ionization (DESI)

DESI is an analytical technique that consists of directly exposing the sample surface to electrically charged microdroplets generated from a suitable solvent. These microdroplets are generated by a pneumatically assisted needle operating under ambient conditions, as shown in Figure 5. On reaching the surface of the sample, the droplets promote the desorption of the analytes from the solid phase into the gas phase, followed by the ionization of these species, allowing their detection by mass spectrometry.^10,22^

**Figure 5:**
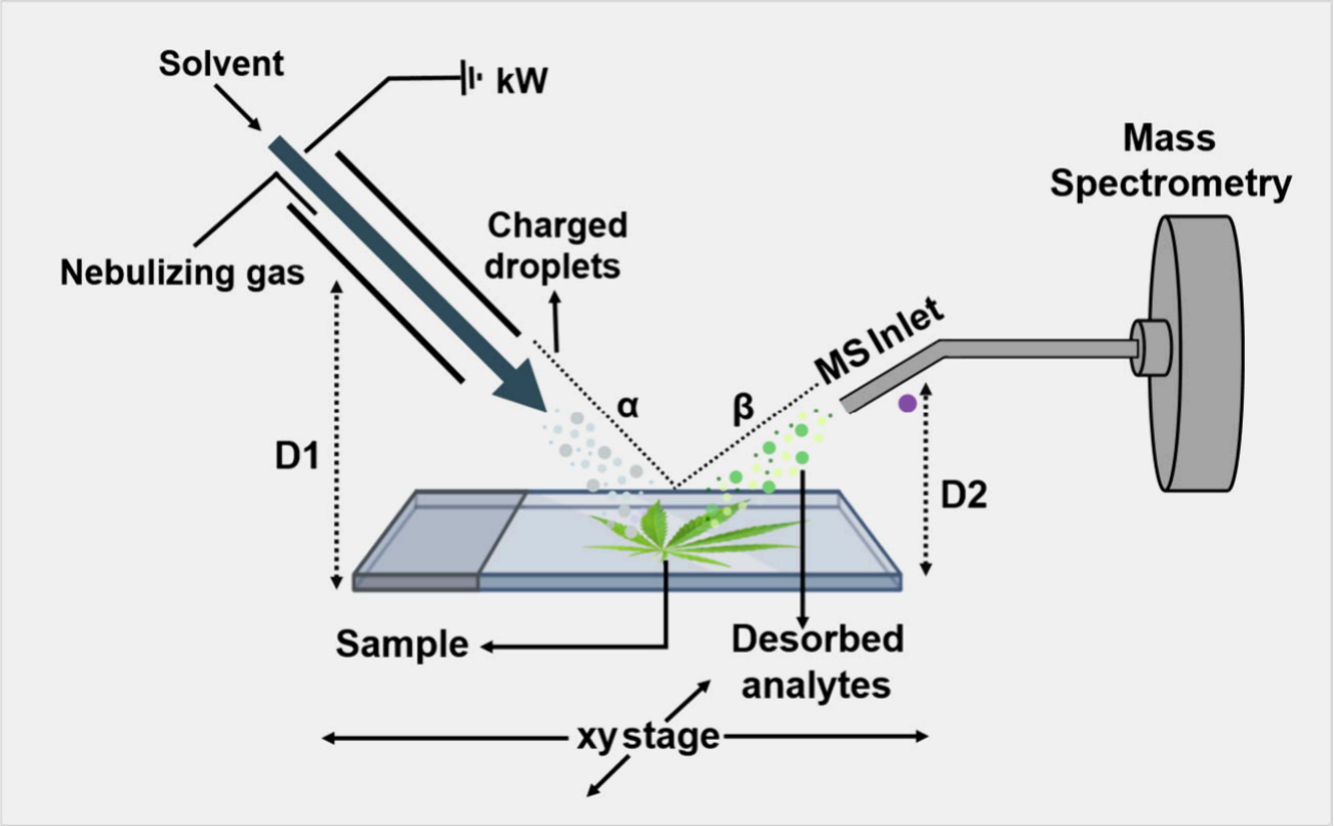
DESI-MS Ionization Schematic representation, analyte desorption process (*Cannabis*) and geometric parameters. Created in BioRender. Santos, N. (2025) https://BioRender.com/gb3f3j1.

In the DESI technique, one of the ionization pathways involves proton transfer mediated by the solvent microdroplets used in the analysis. In this mechanism, hydronium ions (H_3_O^+^) or clusters of water molecules present in the system act as charge transfer agents, promoting the ionization of the analyte molecules adsorbed on the sample surface. As a result, ions are formed in the gas phase, which are then directed to the mass spectrometer for detection and analysis.^23,24^

Recent studies have provided a deeper understanding of the mechanisms responsible for desorption and ionization by using techniques such as DESI mass spectrometry. Various analytical methods, including experimental analyses of microdroplet behavior,^25^ detailed computational modeling of the fluid dynamics involved,^26^ and rigorous evaluations of the effects of surface electrical charges, have contributed significantly to this understanding.

Among the mechanisms proposed to explain the desorption and ionization processes in DESI, the so-called ″droplet capture″ stands out. In this mechanism, a thin liquid layer is first formed on the surface of the sample, which promotes the extraction of the analyte from the solid to the liquid phase.^22^ The analyte is then incorporated into the microdroplets generated during the analysis and transported to the mass spectrometer in the form of a secondary spray. During this process, gradual evaporation of the solvent contained in the droplets produces the dry ions of the analyte through simple acid–base interactions. Due to the broad experimental support and consistency of this model described in the peer-reviewed literature, this mechanism is often indicated as predominant in practical applications of the DESI technique.^27–29^ In addition, these studies have significantly contributed to the optimization of the method’s operating parameters, increasing the efficiency and analytical scope of this technique.^20^

In addition to the above process, there is also a mechanism that occurs through rapid chemical reactions directly in the gas phase. In this case, the ions formed by the solvent rapidly transfer their charges to the desorbed analytes, resulting in an efficient ionization. In addition, another mechanism known as ″chemical sputtering″ is described in which energetic ions collide directly with the surface of the sample.^30^ These collisions promote the release of secondary ions, allowing a complete spectrum to be obtained during analytical scanning by the mass spectrometer.^31^ Complementary studies indicate that these mechanisms, although secondary, are critical to a fuller understanding of the different behaviors observed experimentally in the DESI technique. Additionally, it is possible to transform the DESI platform in an imaging source, commonly named DESI imaging. It consists of sequentially scanning the sample, first along the *x*-axis, where each line analyzed produces a mass spectrum. The platform moves along the *y*-axis, allowing the continuous acquisition of spectra in different regions, resulting in a matrix of pixels that make up the final image. The spatial resolution, typically between 150 and 250 μm, is directly related to the spacing between the pixels.^31,32^

To ensure desorption efficiency and image fidelity, the sample must have a smooth and regular surface.^33^ The geometry of the system also significantly affects the analytical performance, with the main parameters being the spray angle of incidence (α), the collection angle (β), and the distances between the capillary, sample, and spectrometer inlet, as shown in Figure 5.^34^

The solvent composition has a direct influence on both the signal intensity and spatial resolution. While methanol/water mixtures are commonly employed, alternative solvents such as acetonitrile, ethanol, and dimethylformamide (DMF) have also demonstrated satisfactory performance.^35,36^ To avoid analytical bias, the scanning speed (SS) must be compatible with the scanning frequency (SF) of the mass spectrometer.^37^ A typical SS value is 166.66 μm s^–1^, based on a pixel size of 200 μm and an SF of 50 scans min^–1^.

The use of slides with a marker (such as rhodamine, *m*/*z* 443.23540 (C_28_H_31_N_2_O_3_^+^)) helps to calibrate the geometry and check the ionization efficiency. Counts of more than 10^5^ ions and variations of less than 15% across the chromatogram are expected.^32^

### 2.2 Direct Analysis in Real Time (DART)

DART stands out from conventional techniques by enabling the ionization of low-molecular-weight compounds directly from the surface of solids or liquids in a gas stream without the need for sample preparation or chromatographic separation. The DART source was patented in 2005, the same year in which the first study using the technique was published.^15^ However, a prototype was already in use by the United States Army as early as 2002.^38,39^

Recognized as one of the most significant advances in mass spectrometry since the development of ESI and matrix-assisted laser desorption ionization (MALDI) techniques, DART-MS allows for rapid, noncontact analyses with minimal interference from high-molecular-weight matrix components. Currently, the use of this technique is rapidly expanding, accompanied by a growing number of scientific publications and analytical applications.^40^

The DART ionization source consists of two distinct chambers (Figure 6), which are responsible, respectively, for inducing the metastable state of the gas, filtering charged and radical species, and heating the gas flow.^41^ Additionally, a voltage is applied at the gas outlet to remove any residual ionized species and clusters formed by metastable gas molecules.^42^ As a result, the gas emitted from the source contains exclusively electronically excited neutral species, which interact with the sample positioned between the source and the mass spectrometer inlet. This interaction enables the direct, real-time analysis of compounds present in the sample, making DART-MS a powerful technique for a wide range of analytical applications.^41,43^

**Figure 6:**
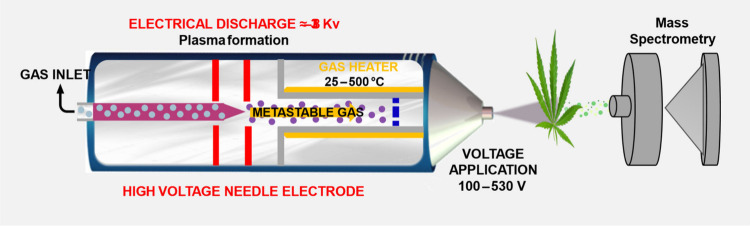
Schematic representation of the DART-MS ionization source, metastable gas formation, and interaction with the sample (*Cannabis*). Created in BioRender. Santos, N. (2025) https://BioRender.com/ey2892k.

The ionization mechanisms involved in the DART-MS technique are highly complex and remain largely underexplored in the scientific literature. Most studies conducted to date have employed helium as the support gas due to its efficiency in generating metastable species. It is assumed that multiple concurrent ionization processes may occur simultaneously, influenced by factors such as the analyte’s proton affinity and ionization potential, its concentration in the sample, the polarity and nature of the carrier gas, as well as the potential presence of auxiliary reagents.^15^ Some authors propose that the ionization mechanisms associated with DART resemble those observed in an atmospheric pressure photo- and chemical ionization techniques.^43,44^

Helium is the most used carrier gas in DART-MS due to the high internal energy of its metastable atoms, which is sufficient to ionize atmospheric water molecules. In positive ion mode, it is generally accepted that the ionization mechanism is initiated by the Penning ionization of water vapor (eq 1), leading to the formation of protonated water clusters (eqs 2 and 3). These clusters subsequently transfer a proton to the analyte, enabling its ionization (eq 4).^43^ When nitrogen or neon are used instead of helium, cation formation predominantly occurs via Penning ionization as well.^15,45^ In this case, the process involves the transfer of energy from a long-lived metastable species (He*) to the analyte molecule (H_2_O), resulting in the generation of a molecular radical cation (H_2_O^+^^•^) and the release of a free electron.

1 $${He}^{*}+H_{2}O\to {H_{2}O}^{+•}+He+e^{-}$$

2 $${H_{2}O}^{+•}+H_{2}O\to {H_{3}O}^{+}+OH^{•}$$

3 $${H_{3}O}^{+}+{nH}_{2}O\to {[\left(H_{2}O{)}_{n+1}+H\right]}^{+}$$

4 $$M+{[\left(H_{2}O{)}_{n+1}+H\right]}^{+}\to {\left[M+H\right]}^{+}+(H_{2}O{)}_{n+1}$$

Alternative source gases, including nitrogen,^46–48^ argon,^49,50^ and air,^51^ have also been investigated, with varying degrees of success. These gases lack metastable atoms with sufficient internal energy to directly ionize water molecules. As a result, direct ionization of the analyte—or of a dopant—is required, making the overall ionization process typically less efficient (eqs 5 and 6).^52^

In such cases, ionization can occur either through Penning ionization of the analyte (eq 5), represented as

5 $$G^{*}+M\to M^{+•}+G+e^{-}$$

where G* denotes the metastable species of the carrier gas (e.g., N_2_* or Ar*), M is the neutral analyte molecule, M^+•^ is the resulting radical cation, G is the neutralized carrier gas, and e^–^ is the ejected electron.

Alternatively, ionization may proceed via proton transfer from a protonated dopant (eq 6), expressed as

6 $$M+D^{+}+[M+{H]}^{+}+D$$

In this equation, M refers to the analyte, D^+^ is the protonated dopant molecule (such as protonated acetone or methanol), [M + H]^+^ is the protonated analyte ion detected by the mass spectrometer, and D is the neutral form of the dopant after proton transfer.

The ionization mechanisms in DART are now well understood, and the Transient Microenvironment Theory proposed by Song et al. (2009) provides a comprehensive explanation of sample matrix effects in both positive and negative ion modes.^53,54^ For nitrogen DART, Song et al. (2018) demonstrated that ionization proceeds mainly through reactions involving NO^+^, a pathway also supported by Cody (2022).^55,56^ In addition, Curtis et al. (2015) used Schlieren imaging to visualize the gas flow dynamics around different shaped objects, revealing how variations in fluid flow can influence ionization and contribute to oxidative processes.^57^ In this context, when air is used as the source gas, reactions involving oxygen and nitrogen species may lead to ozone formation, which can have a detrimental effect on the ion source hardware.

### 2.3 Analytical Potential: Advantages and Limitations of AMS Techniques

Ambient ionization techniques represent a significant advance in mass spectrometry, allowing direct analysis of samples under atmospheric conditions with little or no prior preparation. In this context, the DART and DESI methods stand out for their high analytical efficiency, wide applicability, and remarkable versatility and are particularly relevant for their growing adoption in forensic investigations. Both approaches provide relevant chemical information nondestructively, quickly and with high sensitivity.^27–29,58^

The main advantage of DART-MS is its simplified operation combined with its ability to provide real-time results. By eliminating the need for complex sample preparation steps, this technique is particularly useful in contexts that require rapid response, such as airports, clinical settings, and emergency scenarios. Its noninvasive approach also helps preserve the original sample, minimizing the risk of cross-contamination - an important consideration in sensitive forensic analysis.^58–60^

Another highlight is its broad analytical applicability. The technique has been shown to be effective in detecting gunshot residue, toxicants, and drugs, even in different states of combustion (fully burned, partially burned, or intact), making it particularly useful in forensic ballistics and crime investigation.^61^ In addition, the method offers high sensitivity, with detection limits in the parts per billion (ppb) range, allowing qualitative screening of illicit substances with high precision.^43,62^

However, this technique has limitations related to the nature of the analytes. Ionization in DART-MS favors volatile and semivolatile compounds, limiting its application to low-volatility or thermostable molecules.^63^ In addition, the efficiency of the analysis can be compromised in highly complex matrices, requiring the use of complementary techniques for a more comprehensive characterization.

On other hand, DESI-MS has been characterized by its broad applicability in various analytical contexts, with an emphasis on complex matrices and delicate surfaces. Its use has been particularly relevant in the analysis of biological tissues,^32,64^ pharmaceutical formulations^65^ and historical pigments,^66^ providing an effective and nondestructive alternative for the detection of compounds directly from the surface of the sample. This analytical versatility contributes to its consolidation as a valuable tool in forensic, clinical, and heritage investigations.

In the clinical context, this technique has enabled real-time chemical mapping of tissues, providing important molecular information for differential diagnosis of pathological conditions.^67,68^ The introduction of portable devices, such as the MasSpec Pen, has further expanded the technique’s potential for in vivo applications.^69^ In addition, DESI-MS has found innovative applications in areas such as the conservation and authentication of cultural artifacts by allowing the analysis of historical dyes without damaging the surfaces being analyzed.^70^

Despite its versatility, DESI-MS has limitations. Sensitivity and resolution can vary depending on the composition of the sample and the analytes being analyzed. Although highly effective for rapid screening, the technique may not provide structural information as detailed as that obtained by conventional methods using chromatographic separation coupled to tandem mass spectrometry.^71,72^

In short, both DART-MS and DESI-MS are powerful tools for ambient mass spectrometry. DART-MS is characterized by its ease of use, analytical speed, and high sensitivity to volatile compounds, while DESI-MS offers greater versatility for surface analysis and is compatible with a wide variety of samples, including biological tissues and cultural materials. The choice of the most appropriate technique must consider the specific characteristics of the analyte, the type of matrix, and the desired analytical objectives. Table 1 presents a detailed comparison between the DART-MS and DESI-MS techniques, considering criteria such as the ionization mechanism, sample preparation, type of ion formed, spatial resolution, sensitivity, robustness, main applications, and limitations.

**Table 1:** Analytical Comparison of DART-MS and DESI-MS: Principles, Applications, and Limitations

**Characteristic**	**DESI-MS**	**DART-MS**
Ionization mechanism	Solvent droplets are charged and transported by a nebulizing gas, colliding with a surface, where they desorb and ionize molecules present in the sample, similarly to the process observed in electrospray ionization (ESI)^73^	An excited gas (He or N_2_) generates metastable species that ionize sample analytes through charge exchange or proton transfer mechanisms^43,74^
Sample phase	Typically solids or surfaces (with a supporting substrate)^75^	Solids, liquids, or gases (without preparation or supporting substrate)^76^
Sample preparation	Requires the sample to be deposited on a flat surface, without pretreatment^77^	Minimal or none^74^
Type of ion formed	Positive and negative modes^78^	Positive and negative modes^43,74^
Operating environment	Open to air, with solvent and nebulizing gas^79^	Open to air, with heated gas^74^
Spatial resolution	High spatial resolution; excellent for MS imaging (150–200 μm, with the development of nanoDESI achieving spatial resolution of 10 μm)^75^	Limited (not designed for imaging, requiring coupling with desorption methods)^80,81^
		Studies with LADI-DART-MS have reported spatial resolutions of approximately 87–150 μm.^82–84^
Sensitivity	High, especially for polar compounds^75^	High, but sample-dependent^74^
Robustness	More complex, requiring precise spray alignment^77^	High robustness; easy handling and sample processing with a wide range of positions, distances, and angles for optimized results^74,80^
Applications	Polymers, pharmaceutical analysis, forensic applications, lipid and protein analysis, and tissue analysis^73,79^	Forensic analysis, food testing, pharmaceutical applications, and quality control^74,85^
Limitations	More sensitive to surface geometry and nature^79^	Lower selectivity for complex mixtures; ionization energy is difficult to control^74^

## 3 Contribution of DESI and DART-MS to Forensic Investigations

Forensic chemistry plays a central role in criminal investigations and is responsible for analyzing chemical substances present in material traces, such as drugs, explosives, paints, gunshot residue, and biological fluids. This field applies the principles of analytical chemistry to obtain information that helps to reconstruct criminal events, identify suspects and clarify the circumstances of crimes.^86^

The introduction of techniques such as DESI-MS and DART-MS has increased the efficiency of forensic chemistry, allowing rapid, sensitive analysis with minimal sample preparation. Combined with statistical tools, these approaches offer greater precision in data interpretation, strengthening the role of forensic chemistry in meeting the needs of justice systems.

Figure 7 illustrates the wide-ranging analytical applications of ambient ionization techniques, such as DESI-MS, encompassing areas such as food safety, pharmaceuticals, environmental monitoring, and clinical diagnostics. These examples underscore the versatility of such ionization sources, particularly in facilitating direct and real-time detection of compounds within complex matrices.

**Figure 7:**
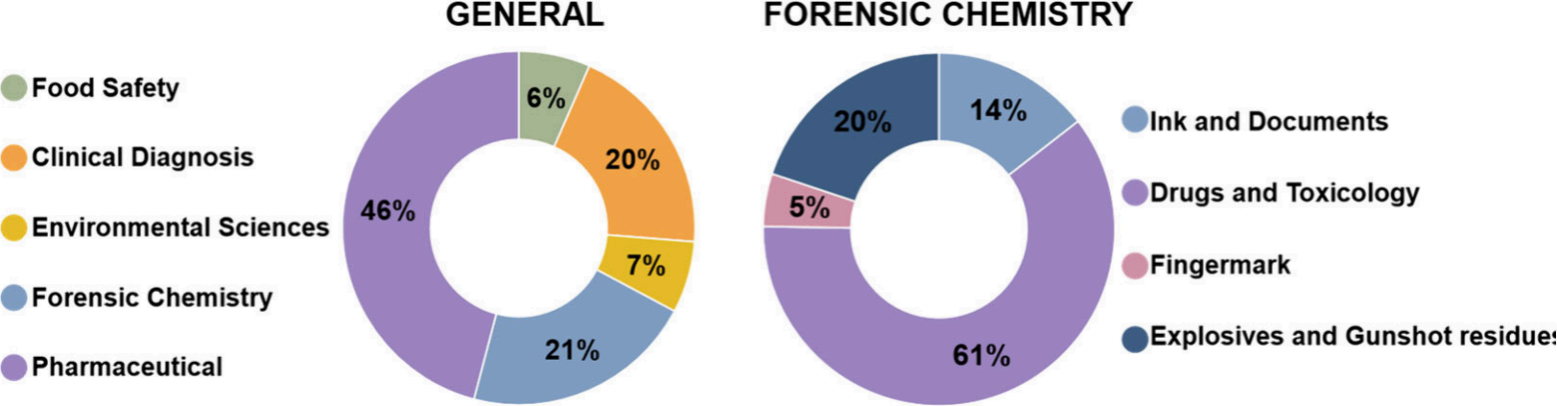
Distribution of DESI-MS applications and its relevance in forensic Chemistry (search terms: “DESI-MS” and “Food Safety; Clinical Diagnosis; Environmental Sciences; Forensic Chemistry; Pharmaceutical”), as well as the main application areas of AMS within the field. Source: Web of Science, accessed on May 02, 2025.

Figure 8, in turn, highlights specific forensic applications, illustrating the successful use of DESI-MS and DART-MS in the analysis of seized drugs, gunshot residues, explosive materials, latent fingerprints, and questioned documents—including the chemical characterization of inks and paper.

**Figure 8:**
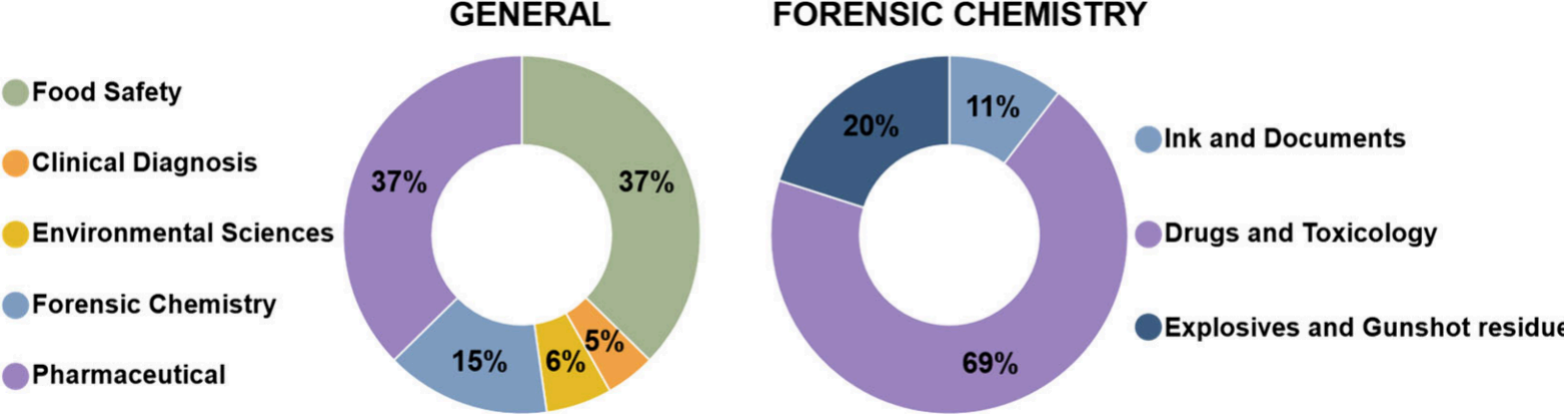
Distribution of DART-MS applications and its relevance in forensic Chemistry (search terms: “DART-MS” and “Food Safety; Clinical Diagnosis; Environmental Sciences; Forensic Chemistry; Pharmaceutical”), as well as the main application areas of AMS within the field. Source: Web of Science, accessed on May 02, 2025.

These findings reinforce the relevance of DESI-MS and DART-MS as powerful tools in forensic science, offering rapid, nondestructive analyses with high sensitivity and minimal sample preparation. Notably, forensic chemistry accounts for a substantial portion of DESI-MS applications (21%) and remains highly significant in DART-MS studies (15%). Within this field, a majority of DART-MS applications focus on drug and toxicological analyses (69%). Importantly, no studies were identified that apply DART-MS to fingermark analysis, highlighting a current gap in the forensic literature regarding this specific application. While DESI-MS demonstrates a more balanced distribution across subareas such as ink and document examination (34%), fingerprint analysis (22%), and explosives and gunshot residue detection (22%). These data underscore the versatility and specificity of ambient ionization techniques in addressing diverse forensic scenarios, particularly in the direct detection and chemical imaging of complex trace evidence.

### 3.1 Analysis of Drugs and Toxicology

The production, trafficking, and counterfeiting of illicit drugs and pharmaceuticals remain significant challenges for forensic and law enforcement agencies. In response, ambient ionization techniques such as DESI-MS and DART-MS have gained prominence in forensic toxicology due to their speed, sensitivity, and ability to analyze diverse sample types with minimal preparation.^74,87^

These techniques are particularly effective in detecting and classifying recreational drugs, which often vary widely in composition and form. Their direct analysis capabilities have been successfully applied to both legal and illegal substances, supporting ongoing efforts to address the growing misuse of psychoactive compounds.^88,89^

In the context of illicit substance detection, the DESI-MS technique has proven effective in identifying compounds such as 3,4-metilenodioximetanfetamina (MDMA), cocaine, flunitrazepam, methamphetamine, cannabinoids, and gamma-hydroxybutyric acid (GHB), which was introduced as a drug of abuse in the early 1990s, even when present in complex matrices such as alcoholic beverages, tissues, and materials of plant origin.^90,91^ Although its application in the analysis of easily manipulated pharmaceutical products is well established, studies have also demonstrated the successful use of DESI-MS for the direct detection of illicit drugs, including MDMA and cocaine.^91–93^ Furthermore, this technique has proven effective in identifying Flunitrazepam (Rohypnol) (a benzodiazepine-class sedative, anxiolytic, and muscle relaxant prescribed for sleep induction - in alcoholic beverages and on surfaces containing dry residues of the substance. The study emphasizes the potential of DESI-MS for quantitative and high-throughput analysis of simulated forensic samples, achieving a limit of quantification of 3 μg mL^–1^ and demonstrating no sample carryover. The method proved to be rapid, sensitive, and selective for detecting spiked beverages typically involved in drug-facilitated crimes, as demonstrated in Figure 9.^90^

**Figure 9:**
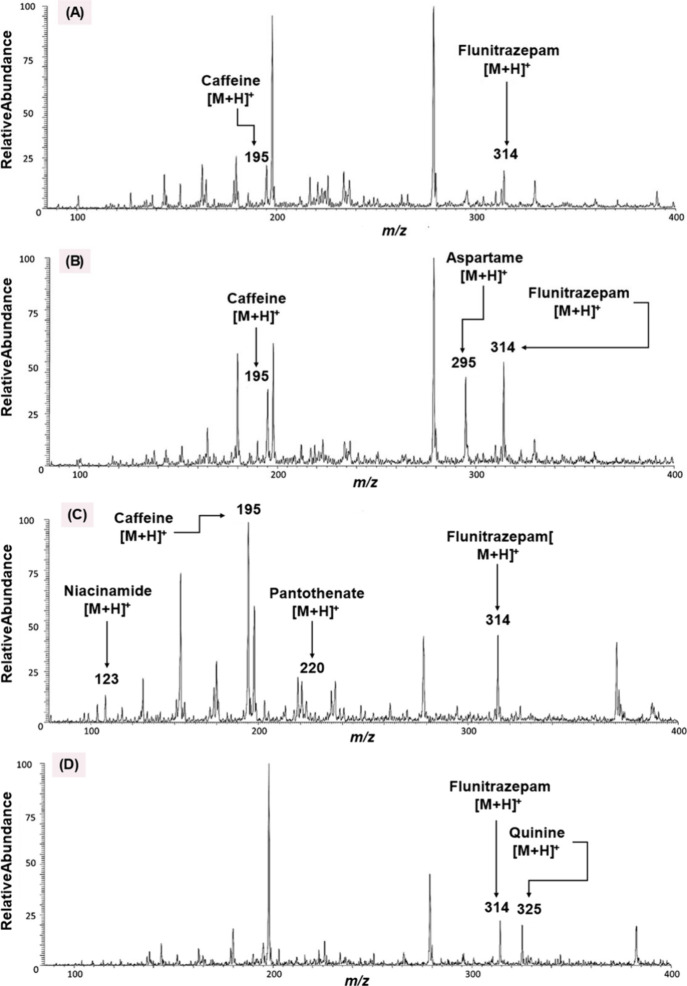
Mass spectra for spiked samples of (A) Kentucky Tavern bourbon and Coca-Cola, (B) Paramount rum and Diet Coke, (C) Korski Vodka and Red Bull, and (D) McCormick dry gin and Canada Dry tonic water. Reproduced with permission from reference 90. Copyright 2012 ELSEVIER.

A significant contribution was the ability to perform analyses directly on the skin or on contaminated surfaces, with good performance even under adverse conditions.^94^ The use of single drop microextraction (SDME) coupled with DESI-MS also yielded positive results in the analysis of methamphetamine in solutions.^89^

Detection of substances in biological matrices is another area in which DESI-MS has been explored. This technique has been used to identify drugs and metabolites in urine, blood and hair with performance comparable to Gas Chromatography–Mass Spectrometry (GC-MS) and Liquid Chromatography–Mass Spectrometry (LC-MS).^95–98^

Despite the need for preparative steps such as solid phase extraction (SPE) in some antidoping applications,^99^ DESI-MS has maintained advantages in terms of speed and analyte discrimination. The use of reactive DESI-MS with hydroxylamine derivatization has allowed efficient detection of anabolic steroids in whole urine,^100^ while protocols using liquid ultrasonic extraction have been proposed for capillary analysis.^101^

In addition to DESI-MS, DART-MS has emerged as a promising tool in the analysis of drugs of abuse and toxicological investigations, mainly due to its ability to perform rapid analyses with minimal sample preparation and in different types of matrices. Pioneering studies,^15^ demonstrated the efficiency of DART-MS in the instantaneous detection of paracetamol in analgesics. Subsequently, Steiner and Larson (2009) successfully applied the technique to the identification of oxycodone, a narcotic analgesic, highlighting the potential of the method for screening in clinical and forensic contexts. Their efforts to compile a database of DART drug spectra provided the foundation for what later became the NIST Forensic DART Database.^102^ Thereafter, Howlett et al. (2011) successfully combined Thin layer chromatography (TLC) with DART-MS to analyze oxycodone with acetaminophen, hydrocodone with acetaminophen, and codeine with acetaminophen, determining the lower limit of detection for each preparation.^103^

Chen et al. (2016) used the DART technique coupled with tandem mass spectrometry on the Q-Orbitrap analyzer to detect traces of illicit drugs without the need for preparatory steps. The method was successfully applied to both standard solutions and real samples, allowing the identification of substances such as p-chloroamphetamine, p-fluoromethamphetamine, GHB, ketamine, methamphetamine, 3,4-methylenedioxypyrovalerone (MDPV), p-methylecatinone, methylone and nimetazepam, Figure 10.^104^ Coon, Beyramysoltan and Musah (2019) explored the use of DART-MS, in combination with chemometric techniques, to generate a database of condom residue spectra that serves as representative fingerprints for forensic analysis.^105^ Similarly, Bridge and Marić (2019) applied DART-MS to the analysis of sexual lubricants at different desorption temperatures (low, high, and pyrolysis) to establish an optimized protocol capable of producing more accurate associations.^106^ Sisco et al. (2019), investigated the potential of trace residues present on the external surface of drug packaging as a viable source for presumptive testing aimed at detecting dangerous synthetic opioids and other emerging psychoactive substances.^107^ Cooman et al. (2021), evaluated 15 drugs of abuse, 15 diluents and 64 mixtures in various proportions to measure bias, precision and repeatability according to United Nations Office on Drugs and Crime (UNODC) guidelines using Raman spectroscopy. A subset of these samples was then analyzed by DART-MS, which correctly identified the analyte present in 92% of cases by library searching.^108^

**Figure 10:**
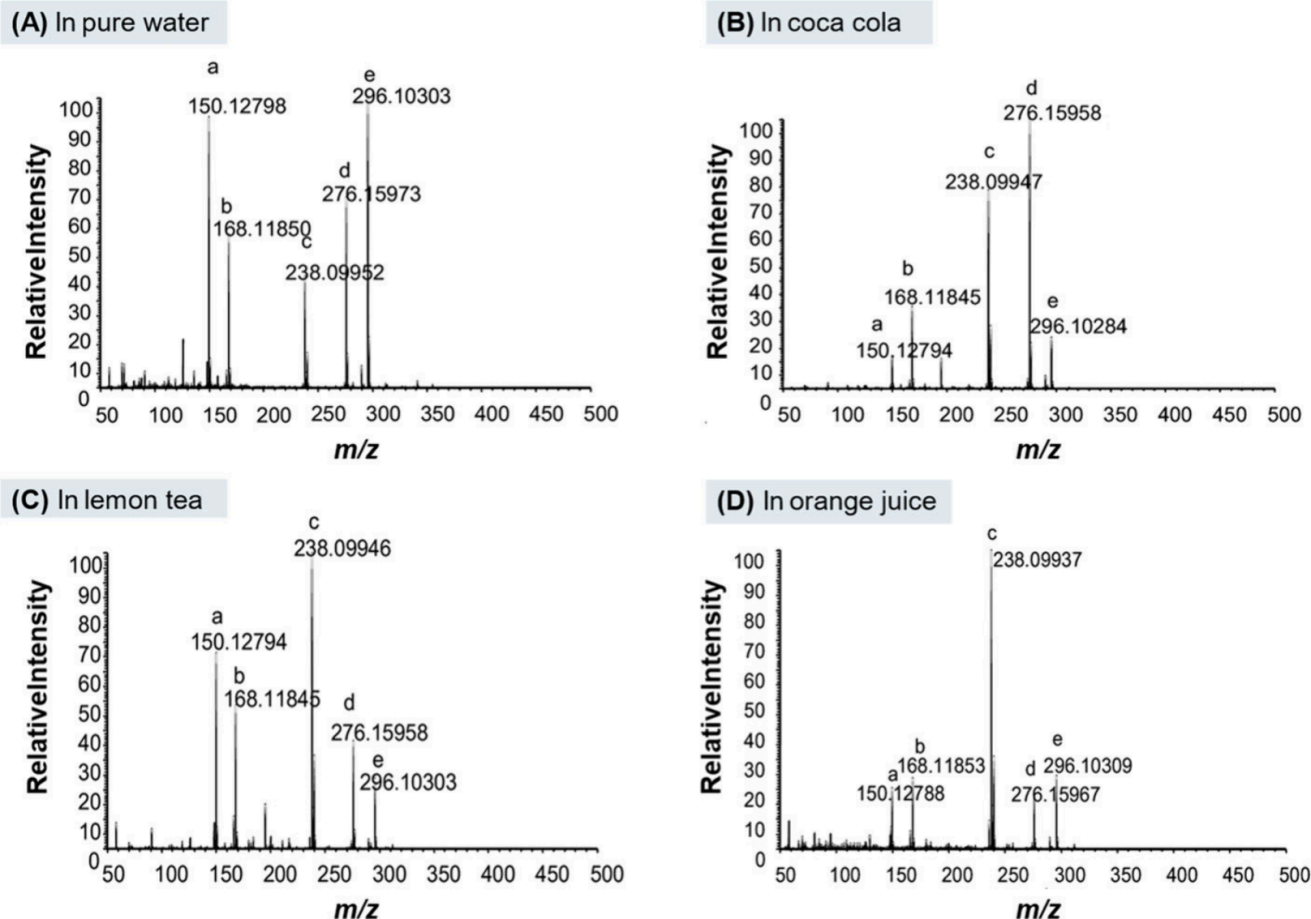
DART mass spectra of the drug mixture in water, cola, lemon tea, and orange juice. The drug mixture contains methamphetamine (a), p-fluoromethamphetamine (b), ketamine (c), MDPV (d), and nimetazepam (e). The concentration of each drug is 83 ppm. Reproduced with permission from reference 104. Copyright 2016 ELSEVIER.

Poklis et al. (2015) reported the identification of synthetic NBOMe compounds directly from impregnated paper using DART-MS in positive ion mode.^109^ Complementarily, Marino et al. (2016) demonstrated that the combination of DART-TOF-MS with nuclear magnetic resonance (NMR) spectroscopy increased selectivity and sensitivity in the detection of synthetic cannabinoids in powder samples, reducing the need for wet chemical processes.^110^ Covey et al. (2025) described the development of a rapid, chromatographic-free screening strategy using DART-MS/MS for the detection of 15 synthetic cannabinoids and their metabolites in urine samples. In addition to stimulants, the technique also proved effective in the analysis of hallucinogens.^111^ Applying both ionization sources, Fernández et al. (2006) employed DESI-TOF and DART-TOF for the rapid screening of counterfeit artesunate tablets (antimalarial), obtaining good signal-to-noise ratios within just 5 s of analysis. The DART analysis also contributed to a multigroup investigation and the identification of at least one illicit laboratory.^112^

Finally, Sisco et al. (2021) described the new NIST DART-MS Forensics Database, formalizing the release of its updated version. Developed to address increasing caseloads and the evolving drug landscape, the updated database incorporates an automated data evaluation process to facilitate interpretation of complex spectra and expand coverage to novel psychoactive substances.^113^

### 3.2 Fingermark Analysis

Fingerprint analysis is a forensic technique used to identify individuals based on the unique patterns and ridges of fingerprints. This method, which has its roots in ancient Babylon, is essential in a variety of industries, including law enforcement, where it helps identify suspects and victims as well as commercial security and biometric authentication applications. Fingerprint patterns are classified into three main types: arches, loops and whorls, each with distinct characteristics that are critical for accurate identification and comparison.^114^

DESI-MS has emerged as a promising technique in the forensic analysis of fingermarks by combining imaging capabilities with chemical detection in a single approach. Among the 110 compounds identified, or tentativepotential for identifying both exogenous compounds (originating from external sources, such as contaminants, drugs, or cosmetics) and endogenous compounds (naturally present in the body, such as amino acids, lipids, and metabolites) within the marks. This enables not only the visualization of ridge morphology but also the extraction of information regarding the individual’s recent activities. With a spatial resolution of approximately 150 μm — sufficient to resolve ridge details — DESI-MS has been successfully applied to fingermarks deposited on various surfaces, including glass, paper, and plastic.^115–117^

In studies conducted by Ifa and collaborators (2008), fingerprints were collected following contact with small quantities (5 mg) of various substances, including drugs of abuse — such as cocaine and Δ^9^-tetrahydrocannabinol (Δ^9^-THC) — and explosives like hexahydro-1,3,5-trinitro-1,3,5-triazine (RDX). The findings demonstrated the efficiency of DESI-MS in detecting forensically relevant compounds at trace levels.^116^ Moreover, endogenous compounds such as palmitic and myristic acids were also identified. Given that the relative abundance of these substances varies between individuals, the technique presents potential for differentiating overlapping fingermarks from distinct donors, thereby enhancing its applicability in forensic investigations.^116^

It is important to emphasize that DESI-MS is not intended to replace traditional fingermark detection methods such as fingerprint powders or chemical reagents. Instead, it serves as a complementary tool that provides additional information beyond the mere visualization of ridge patterns. For instance, it enables the inference of prior contact with illicit substances and, according to some reports, may even allow the assessment of ethnic characteristics based on the chemical composition of the secretions present in the fingermark.^117^

Desorption electrospray ionization mass spectrometry (DESI-MSI) was employed to perform chemical and spatial analyses of the latent fingerprints. To simulate the lipid composition typically found in fingerprints, samples were collected by swiping a glass slide across the foreheads of consenting individuals. The application of a machine learning algorithm — Gradient Boosting Decision Tree (GBDT), enabled the classification of donors based on gender, ethnicity, and age group (within a 10 - year range), achieving accuracy of 89.2%, 82.4%, and 84.3%, respectively. Key chemical markers identified through GBDT feature selection were further characterized by tandem mass spectrometry. As a proof of concept, the trained model was successfully applied to overlapping fingerprints from multiple individuals, accurately predicting gender and ethnicity. These findings underscore the potential of integrating DESI-MSI with machine learning approaches to enhance forensic fingerprint analysis.^118^

The study conducted by Frisch et al. (2024) represents a significant advancement in the field, reporting for the first time the application of DESI-MS for the chemical analysis of fingermarks collected using gelatin lifters—a technique commonly employed at crime scenes, as illustrated in Figure 11.^119^ The imaging could be performed directly on the gelatin substrate without the need for further sample preparation, supporting the immediate operational use of the method for the collection of fingermarks from crime scenes. The technique was validated on fingermarks enhanced with conventional forensic powder and lifted from various substrates, including glass, stainless steel, painted aluminum, polystyrene, cardboard, and plastic, showing reliability even in marks that had been transferred multiple times.^119^

**Figure 11:**
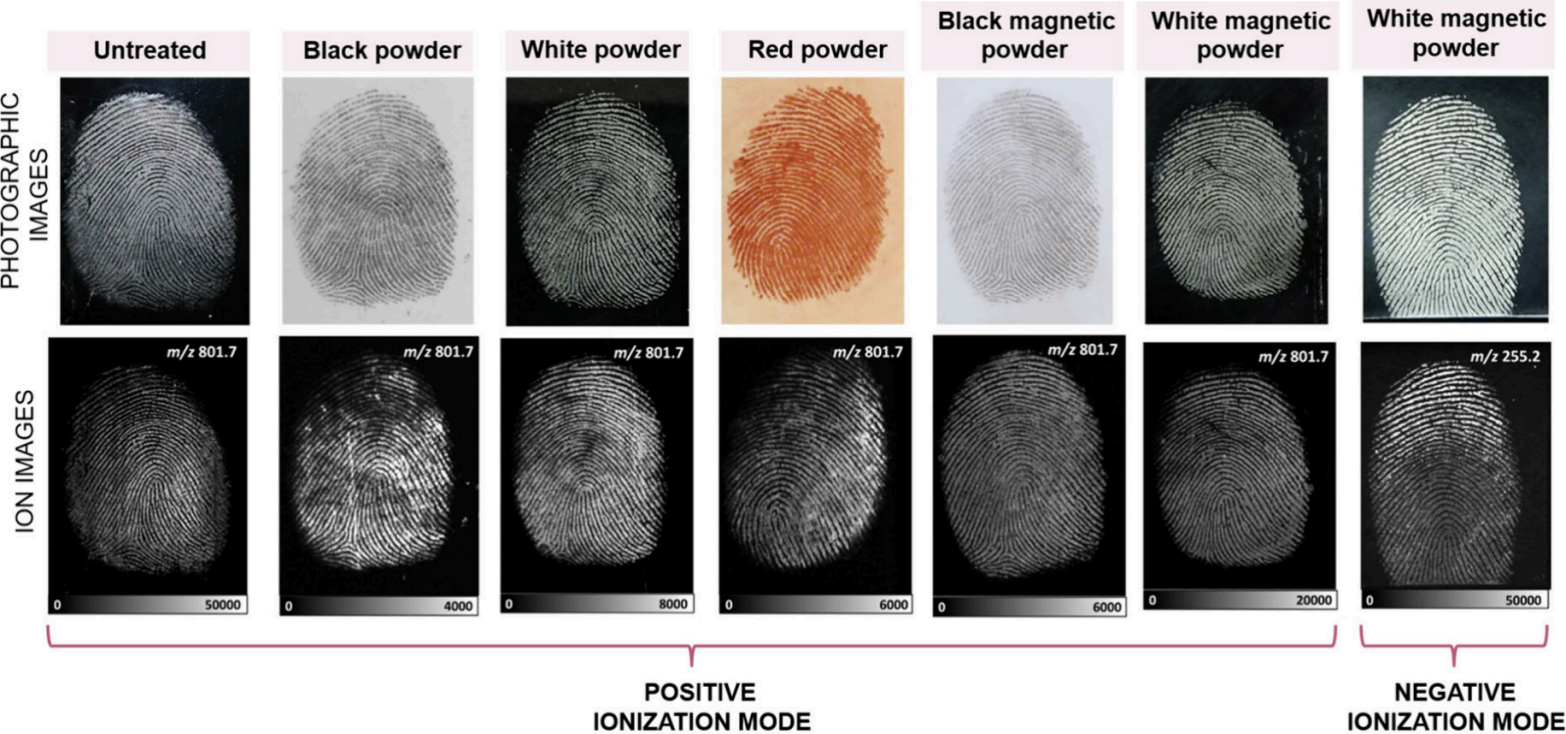
DESI-MS ion images of sebum-enriched fingermarks from the same person lifted with gelatin lifters after treatment with different forensic dusting powders. For comparison, an image of an untreated sebum-enriched fingermark lifted with a gelatin lifter is also shown. The corresponding linear intensity scale is shown below each ion image. Photographic images of the samples taken prior to DESI-MS imaging are shown in the top row. Reproduced with permission from reference 119. Copyright 2024 American Chemical Society.

Thus, although DESI-MS emerges as a promising and complementary tool for the chemical and visual analysis of latent fingermarks, its application in this area remains relatively limited in the current literature. In contrast, MALDI-MS has been more widely explored for such purposes, demonstrating greater consolidation in the field.

To date, no published studies have reported the use of DART-MS specifically for the analysis of latent fingermarks. Nonetheless, the integration of emerging techniques like DESI-MS into traditional forensic workflows holds potential to enhance the scope and depth of fingermark analysis, contributing meaningfully to criminal investigations and the scientific understanding of dermal secretions.

### 3.3 Analysis of Inks and Documents

Forensic analysis of dyes and pigments is critical to solving crimes, especially when these compounds are present in traces on documents, clothing, or surfaces associated with a crime scene. These colored substances, such as dyes, pigments, and paints, are visible through interaction with light and can establish a link between the suspect and the crime scene. As is characteristic of trace evidence, these materials are present in limited quantities; however, such amounts are typically sufficient for analytical procedures, enabling comparisons with reference samples and contributing to the individualization of forensic evidence. Pigments are also used for control and quality assurance in investigations.^120,121^

In the field of mass spectrometry, DESI distinguishes itself by enabling the simultaneous acquisition of chemical and spatial information directly from sample surfaces. Van Berkel and Kertesz (2006) employed an automated DESI ion source to generate images of analytes on thin-layer chromatography (TLC) plates and forensic documents. The method achieved a spatial resolution of 400 μm, allowing for the differentiation of various ink types within a single text.^122^ Despite the high quality of the images obtained, the technique is associated with extended analysis times and, in certain cases, necessitates the use of multiple solvents to effectively discriminate between inks. Optimal selection of solvent type and flow rate is crucial, as inadequate parameters may lead to undesirable ink dispersion on the analyzed surface.^122,123^

Ifa et al. (2007) demonstrated the applicability of DESI-MS in the forensic analysis of documents by enabling the direct characterization of ballpoint pen inks on paper without the need for prior sample preparation. The technique produced 2D molecular images and proved effective in the detection of document forgeries, offering notable advantages in terms of analytical speed and preservation of the original material. Inks containing the dyes Basic Blue 7 (as shown in Figure 12), Basic Violet 3, and Solvent Blue 26 were evaluated, and it was observed that the solubility of these analytes had a direct impact on desorption efficiency.^124^ Khatami et al. (2008) applied DESI-MS in combination with TLC and ESI-Orbitrap to detect counterfeiting of thermochromic ink pens. Marker ions (*m*/*z* 400, 405, 615, and 786) were identified in the invisible state of the ink and served as chemical indicators of tampering. The technique proved to be effective, rapid, and minimally invasive for forensic document analysis.^125^

**Figure 12:**
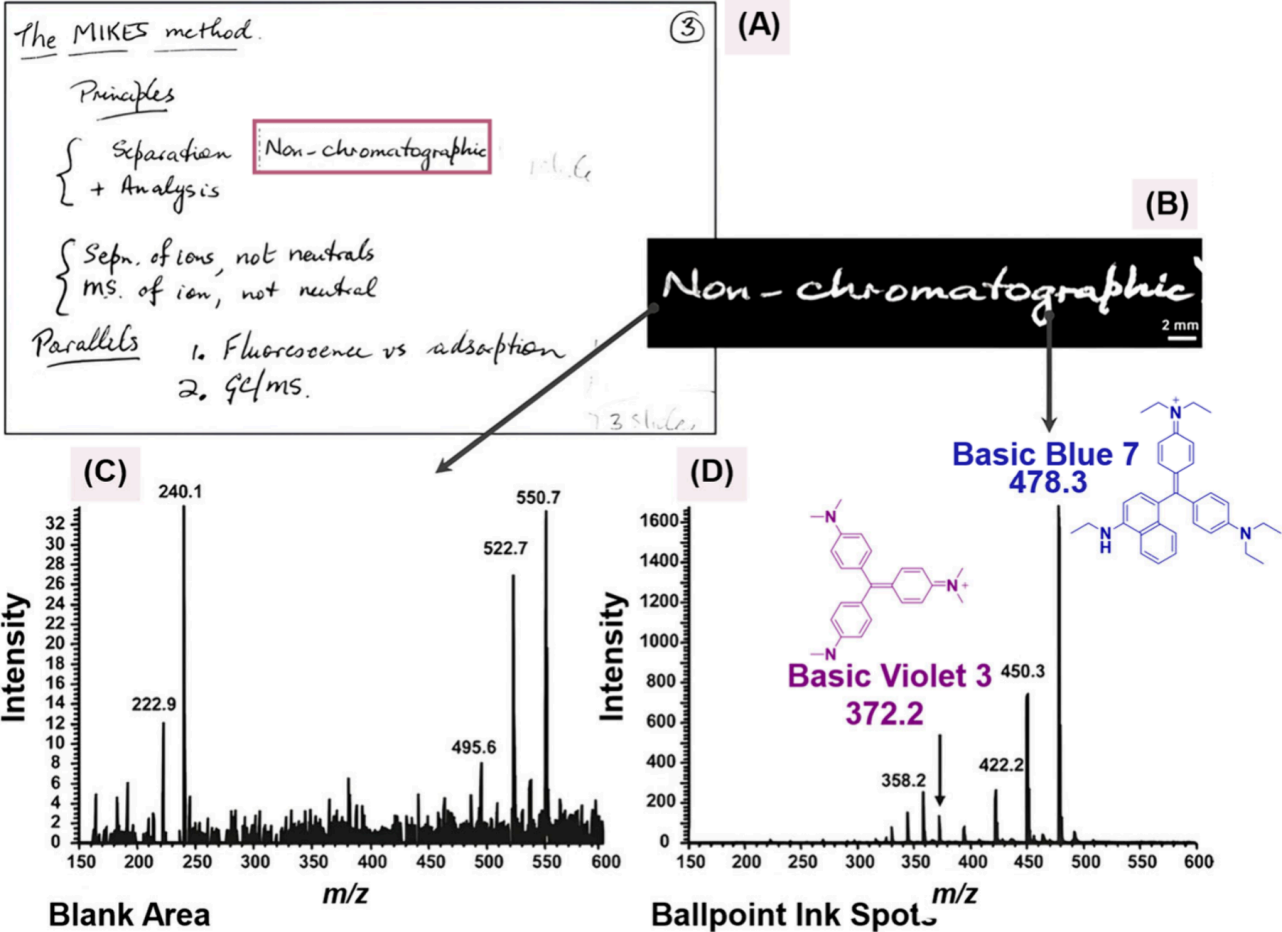
(A) Optical image of an index card from 1981; (B) two-dimensional DESI image of Basic Blue 7 (*m*/*z* 478) in the highlighted area; (C) full scan mass spectrum of a blank spot; and (D) full scan mass spectrum of the written area. Reproduced with permission from reference 124. Copyright 2007 Royal Society of Chemistry.

Sun et al. (2022) evaluated the use of DESI-MS to differentiate ink entries based on their age. The technique identified changes in compounds such as dyes and polyethylene glycol derivatives as well as a reduction in surfactants following aging, enabling the distinction between fresh and aged inks without damaging the document. The results demonstrate the potential of DESI-MS for the relative dating of documents.^126^

Braga et al. (2023) proposed a rapid and straightforward method for dating pen inks in documents by combining transform infrared attenuated total reflectance (FTIR-ATR), DESI-MS, and multivariate modeling. The study analyzed documents dated from 1960 to 2022, identifying chemical variations in dyes, such as Basic Violet 3. The fusion of spectral data resulted in a predictive model with a margin of error of ± 5 years, validating the approach as a promising tool for forensic document dating.^127^

The following year, Yuan et al. (2024) investigated the composition of gel pen inks using DESI-MS to differentiate commercial brands and models. A total of 227 commercial samples were analyzed, and the resulting data were processed by using statistical methods such as the K-means algorithm. The results confirmed the effectiveness of DESI-MS as a minimally destructive analytical technique capable of identifying variations in chemical composition between different models of the same brand, as well as similarities between different brands.^128^

Subsequent studies recommend short sampling times, less than 10 s, to reduce the risk of document damage and to optimize signal intensity.^117^ Furthermore, the use of mobile platforms with DESI-MS allows on-site analysis, provides position-specific mass spectra, and favors a nondestructive approach. Considering these characteristics, the technique shows promise not only as a complement, but also as a potential replacement for the traditional sequence of document ink analysis, as it allows the composition and location of different inks to be determined in a single acquisition.^117^

In parallel with the development of DESI-MS, DART-MS was used in the analysis of pigments and paints. One of the main advantages of DART-MS is its ability to detect organic compounds and pigments directly on the sample, without the need for prior preparation.^42^ Highlighted the advantage of the technique as it is nondestructive, which makes it suitable for sensitive forensic samples, such as documents or fragile traces.

Ink analysis employing DART-MS enables the rapid and nondestructive generation of chemical profiles directly from questioned documents without altering their visual integrity, as described by Jones et al. (2013). The method has been effectively applied to ballpoint, gel, and fluid writing inks across various paper substrates, presenting minimal interference, except for instances involving heavier or extensively processed papers. Time-dependent investigations revealed significant alterations in ink spectra within the initial months following application, attributed to the evaporation of volatile constituents with subsequent spectral stabilization. A library search conducted on 166 well-aged inks confirmed the robustness of the technique, achieving a notable identification success rate of 92%.^129^

In this context, Lago et al. (2016) applied direct analysis in real time high-resolution mass spectrometry (DART-HRMS), GC-MS, and ultrahigh performance liquid chromatography electrospray ionization high-resolution mass spectrometry (UHPLC/ESI-HRMS) to investigate printing-related contaminants present on both the printed and food-contact surfaces of undercured food packaging. Among the 110 compounds identified or tentatively identified, 35 were associated with printing processes, and 28 showed evidence of setoff, a phenomenon in which ink or other printed material unintentionally transfers from one surface to another during storage or handling. The study confirmed the presence of 16 photoinitiators, seven scission products, five probable photoinitiator degradants, and at least five novel contaminants, including 4-morpholin-4-yl-benzaldehyde and 3-phenyl-2-benzofuran-1­(3H)-one.^130^

Chen and Wu (2017) screened organic pigments in automotive paints by FTIR and used DART-MS to confirm the results. This approach eliminated the need for complex extraction or separation processes, highlighting the efficiency of the technique for field application.^131^ In 2021, Sisco and Forbes reinforced the versatility of DART-MS by demonstrating its ability to detect not only pigments, but also additives and other components present in various matrices.^61^

Complementing these applications, Williamson et el. (2016) demonstrated that it is possible to differentiate inkjet printer inks, even from cartridges of the same manufacturer, based on the composition of semivolatile polymers, highlighting the importance of sensitive techniques in characterizing similar materials.^132^ More comprehensively, Trejos et al. (2016) created an ink database using different instrumental techniques and the MassHunter Workstation software (v. B.05.00, Agilent, USA), contributing to the standardization and comparison between samples. A searchable database comprising 319 ink samples from toner, inkjet, offset, and intaglio printing sources was developed to enhance the chemical analysis of ink evidence. Data were generated using five analytical techniques, FTIR, scanning electron microscope and energy dispersive X-ray spectrometer (SEM-EDS), laser ablation inductively coupled plasma mass spectrometry (LA-ICP-MS), DART-MS, and pyrolysis gas chromatography–mass spectrometry (Py-GC-MS) — and processed using a partial least-squares discriminant analysis (PLS-DA) algorithm to calculate similarity scores for ink association. The effectiveness of each method varied by ink type, with LA-ICP-MS yielding the best results, followed by SEM-EDS and DART-MS, while FTIR and Py-GC-MS were more suitable for classification. Combining data from complementary methods, such as LA-ICP-MS and DART-MS, further improved ink discrimination and classification.^133^

Williamson et al. (2017) evaluated the application of DART-MS in the characterization of ink intersecting lines (crossline intersections), comparing its performance with that of other analytical techniques. Luminescent compounds, such as crystal violet and methyl violet, were identified in mixtures in the ink formulations by all methods used, including DART-MS. Although this technique proved effective in detecting these components, none of the approaches were able to determine the order of ink deposition in the overlapping areas.^134^

Drury et al. (2018) compared two ambient ionization techniques coupled to a JEOL AccuTOF MS: Direct sample analysis-mass spectrometry (DAS-MS) (PerkinElmer) and DART-MS (IonSense). Both techniques were found to be effective in the analysis of writing inks and produced similar spectra. DSA-MS exhibited lower background signals as a result of its closed-source configuration, whereas DART-MS, employing an open-source design, provided greater flexibility in sample positioning. This allowed for the analysis of smaller fragments with optimized sensitivity, as illustrated in Figure 13.^135^

**Figure 13:**
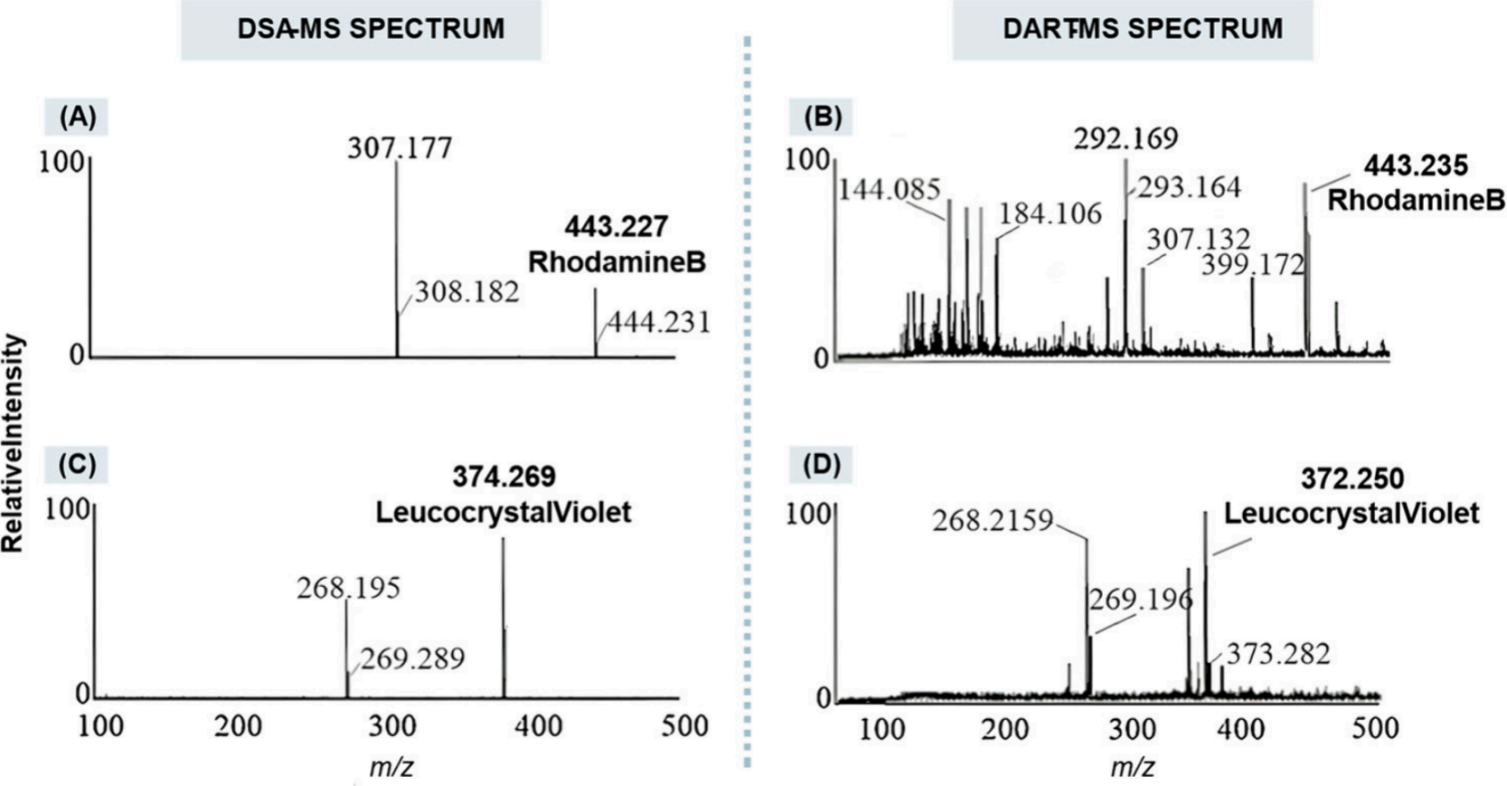
Analysis of gel pen and ballpoint pen by DSA-MS and DART-MS. (A) DSA-MS spectrum of gel pen; (B) DART-MS spectrum of Zebra gel pen; (C) DSA-MS spectrum of blue ballpoint Paper Mate stick pen; (D) DART-MS spectrum of blue ballpoint Paper Mate stick pen. Reproduced with permission from reference 135. Copyright 2018 ELSEVIER.

Marić et al. (2018) characterized automotive paints from four vehicles using DART-TOF-MS and a standard pyrolysis coupled GC-MS (py-GC-MS) protocol. Principal Component Analysis (PCA), a multivariate statistical technique commonly used to reduce dimensionality and highlight patterns in complex data sets, was applied to the resulting data. The analysis demonstrated that both techniques displayed discriminatory power comparable to that of the analyzed samples. However, in some cases, DART-TOF-MS identified compounds not detected by py-GC-MS, and vice versa, suggesting that these methods can provide complementary information in the forensic analysis of automotive coatings.^136^

The study by Zhang et al. (2022) presents MSI as an effective visual tool for ink differentiation and introduces an innovative approach for ink source prediction based on DART-MS spectral data. A total of 106 ink samples from three major printer brands — Canon, Epson, and HP—were analyzed. To extract key spectral features, dimensionality reduction techniques such as PCA, non-negative matrix factorization (NMF), and probabilistic latent semantic analysis (pLSA) were employed. These features were subsequently visualized through MSI. A convolutional neural network (CNN) trained on the reduced data achieved high predictive accuracy, reaching 100% for black, magenta, and yellow inks and 99.6% for cyan. Results from blind tests confirmed that expanding the MSI acquisition area improves predictive performance, reinforcing the recommendation for broad-area ink detection in forensic analyses.^137^

Chen et al. (2023) proposed a method for predicting the source of black inks using direct analysis in DART-MS, in combination with dimensionality reduction techniques and likelihood ratio (LR) analysis. A data set consisting of 39 chemically similar inks from three major manufacturers was evaluated. Dimensionality reduction was performed using PCA and uniform manifold approximation and projection (UMAP), with UMAP demonstrating superior performance in distinguishing intra- and intercluster variations. The predictive model achieved an accuracy of 99.83%, based on more than 41,000 spectra. LR calibration, conducted using the pool-adjacent-violators algorithm and logistic regression, yielded excellent equal error rates (0.004), with only minor differences in misleading evidence rates and log LR cost metrics. Blind testing further validated the robustness of the proposed approach, reinforcing its applicability in forensic ink source attribution.^138^

Therefore, both DESI-MS and DART-MS have established themselves as relevant analytical tools for the characterization of paints and pigments in forensic contexts. While DESI-MS allows direct spatially resolved analysis on documents, DART-MS offers a fast, sensitive, and nondestructive alternative for screening and confirmation of compounds. The combined use of these techniques represents a significant advance in the examination of chemical evidence, increasing the reliability of the results and the efficiency of the chain of custody.

### 3.4 Analysis of Explosives and Gunshot Residues

Explosives and Gunshot Residue (GSR) detection plays a critical role in forensic science, helping to reconstruct criminal events. Explosives are highly reactive substances that release large amounts of energy in the form of light, heat, sound and pressure when triggered.^139^ Explosives release stored chemical energy violently, causing significant destruction. GSR, formed by the combustion of propellants during firearm discharge, has a variable composition influenced by the ammunition type and barrel residues. Forensic analysis of GSR helps determine critical details in investigations, including shooting distance, weapon identification, the shooter’s involvement, and the nature of the incident.^140^

The DESI-MS technique has been widely employed in the analysis of explosive compounds, particularly nitro-organic explosives such as RDX, C-4, Semtex-H, and Detasheet.^141^ Several studies have demonstrated the feasibility of this technique on a range of surfaces—including glass, paper, plastic, and metal, as well as its capability for direct detection on skin and textile substrates. Figure 14 specifically depicts the analysis performed on garment fabric.^97,142^ Moreover, the successful analysis of latent fingerprints contaminated with residues of these compounds was also been reported.

**Figure 14:**
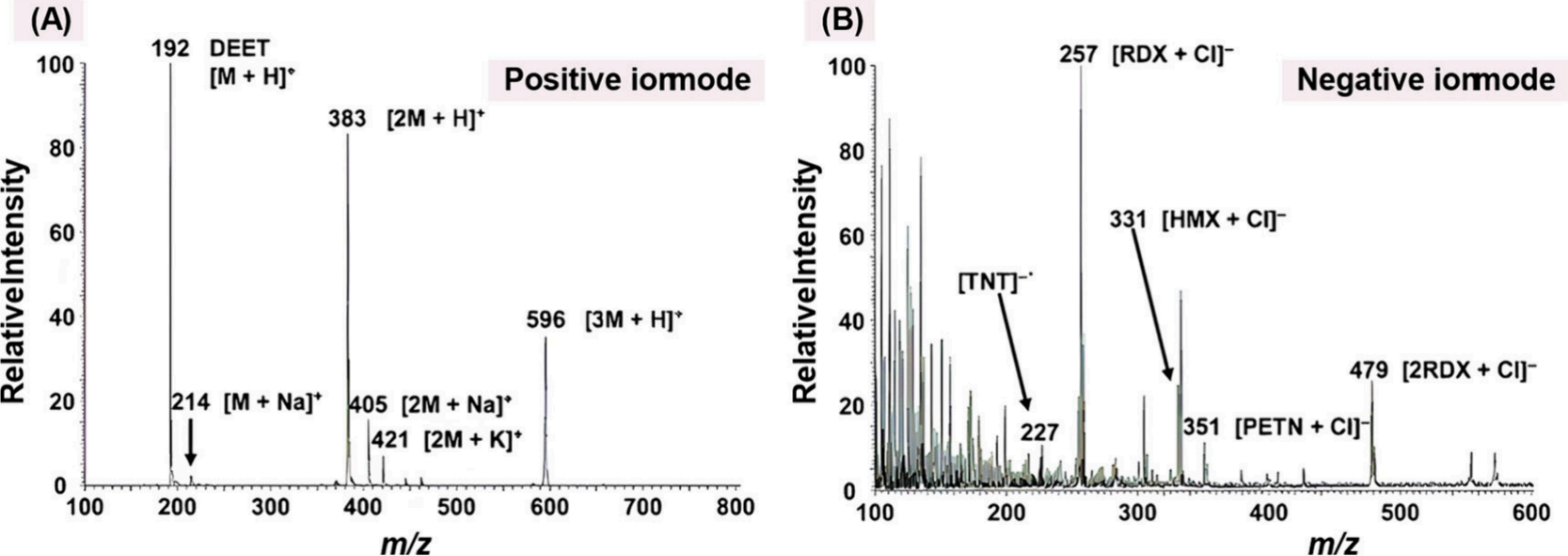
DESI mass spectrum of 100 ng mixture of Trinitrotolueno (TNT), RDX, cyclotetramethylene tetranitramine (HMX) and pentaerythritol tetranitrate (PETN) on cotton fabric in the presence of N,N-diethyl-m-toluamide (DEET), showing the results in both (A) positive ion mode and (B) negative ion mode. Reproduced with permission from reference 97. Copyright 2008 Royal Society of Chemistry.

DESI-MS exhibits variable sensitivity depending on the analyte and surface, with detection limits typically in the picogram range and reaching femtogram levels for TNT on nonporous surfaces. The technique has been effectively applied using different mass spectrometers, especially in negative ion mode.^143^ Sensitivity can be further improved through reactive DESI strategies utilizing chloride-doped solvents, which markedly enhance the ionization efficiency of explosive compounds such as RDX and HMX. When combined with the intrinsic advantages of DESI—namely, its ability to perform rapid and direct analyses from various surfaces without sample preparation, achieve subnanogram detection limits, and provide high specificity confirmed by tandem mass spectrometry—this approach reinforces the technique’s applicability for trace-level detection of explosives in both simple and complex forensic matrices.^141^

Cotte-Rodriguez et al. (2006) expanded their investigations to include the analysis of organic peroxide explosives such as triacetone triperoxide (TATP), which were detected in the form of alkali metal complexes, with limits of detection (LOD) in the nanogram range.^144^

Homemade micro-SPE cartridges with different adsorbent materials were evaluated for the DESI-HRMS analysis of soil samples containing explosives such as TNT, RDX, HMX, PETN, and trinitrophenylmethylnitramine (Tetryl). The method achieved quantitation limits in the low nanogram per kilogram range, confirming its suitability for ultratrace detection. In addition to reducing costs and enabling high-throughput analysis through minimal sample preparation, the approach demonstrated enhanced extraction efficiency, with DESI-HRMS responses at least five times greater than those obtained from conventional solid–liquid extracts spotted onto politetrafluoretano (PTFE) slides.^145^

In addition to explosives detection, DESI-MS has also been applied to the analysis of GSR, particularly with the recent introduction of heavy metal-free primers.^146–148^ While most analytical approaches focus on the identification of inorganic residues, studies have shown that ambient sources such as paint and fireworks can produce particles with similar characteristics. Therefore, there is a growing need for more comprehensive analytical methods capable of detecting and characterizing organic compounds present in the gunshot residue (OGSR).

In this context, the DESI-MS technique demonstrated efficiency in detecting degradation products of stabilizers such as diphenylamine, as well as compounds like methyl centralite and ethyl centralite, including in skin samples, without the need for prior sample preparation.^146,149^ Zhao et al. (2008) developed a rapid and sensitive method—with an analysis time of less than five seconds—for the identification of these compounds.^149^ Additionally, Morelato et al. (2012) applied DESI-MS directly to adhesive stubs used in SEM-EDX analyses, without causing significant interference in subsequent results.^146^

The combined use of DESI-MS and SEM-EDX enables the simultaneous identification of both organic and inorganic residues within a single sample, thereby enhancing their evidentiary potential in forensic analyses. Nonetheless, the method still presents limitations in sensitivity, particularly for the detection of specific compounds, such as nitroglycerin and nitrocellulose. To address this issue, Venter et al. (2010) proposed strategies involving the preconcentration of residues on more suitable surfaces prior to analysis, which may contribute to overcoming these analytical challenges.^150^

DART-MS has emerged as an innovative and efficient analytical tool within the field of forensic science, notably due to its ability to perform rapid and presumptive screenings directly on a wide range of surfaces such as metals, glass, wood, adhesive tapes, and polymers. However, the effectiveness of this technique may be influenced by certain factors, including the porosity of the substrate—which tends to retain the target analyte, thus hindering its release into the gas phase—and the thermal properties of the explosive compounds, which directly impact the desorption area.^61^

The flexibility of DART-MS and its variants has enabled the detection of a broad spectrum of organic explosives, including dinitrotoluene, trinitrotoluene, trinitrobenzene, nitroglycerin, PETN, RDX, and HMX. Considering the constant emergence of new explosive compounds on the market, alternative sample introduction techniques, such as thermal desorption DART-MS, Quick Strip, and ionRocket, have been developed to overcome the limitations associated with conventional methods.^74^

Recent advances have highlighted the effectiveness of DART-MS when combined with complementary techniques, such as Raman spectroscopy, enabling the generation of orthogonal signatures capable of precisely distinguishing between explosives, binders, plasticizers, and additives.^151^ The integration of an infrared thermal desorption module (IRTD-DART-MS) has further enhanced the analytical performance by creating specific thermal profiles for different compounds, allowing them to be desorbed at optimal temperatures.^152^ This approach has also proven effective in detecting inorganic constituents, such as potassium perchlorate in flash powder, as well as potassium nitrate and sulfur found in black powder.^153^

In terrorism-related scenarios, where explosive materials may be present in trace amounts, the high sensitivity and speed of DART-MS are essential for effective postblast residue screening. The combination of DART with high-resolution Orbitrap MS enables rapid analysis of energetic formulations including complex plastic explosives. Through Kendrick mass defect analysis, polymeric components such as polyisobutylene, polybutadiene, and polystyrene were identified in samples such as PG2 and Semtex 10. Postblast residues showed increased unsaturation and reduced oxygenation, as revealed by principal component analysis, demonstrating the technique’s effectiveness in characterizing chemical changes in explosives after detonation.^154^

In addition to its application in the detection of explosives, DART-MS has shown notable effectiveness in the analysis of GSR, providing valuable contributions to forensic ballistic investigations, as illustrated in Figure 15. The technique has been successfully employed in the detection of components from propellants, commercial ammunition, and improvised explosive devices, covering residues at various stages of combustion — from unburned to completely combusted material. This versatility has supported the resolution of key forensic questions, such as identifying the type of ammunition used, estimating the shooting distance, and establishing a potential link between the suspect and the discharged firearm.^155^

**Figure 15:**
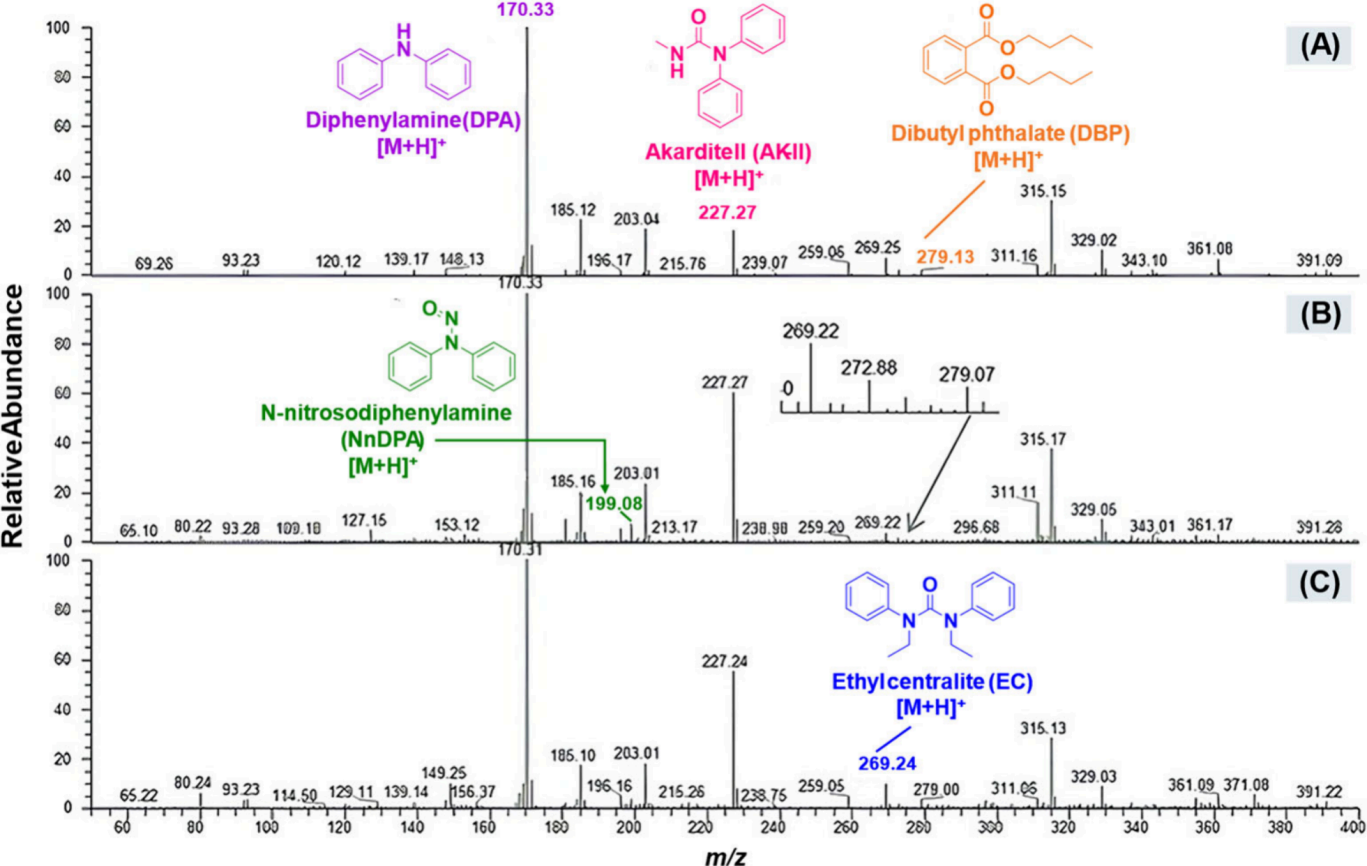
Representative positive ion mass spectra of Hodgdon Lil’ Gun smokeless powder at (A) 148 °C, (B) 160 °C, and (C) 172 °C with a constant flow rate of 3 L min^–1^ and a sampling time of 5 min. Reproduced with permission from reference 155. Copyright 2016 ELSEVIER.

Black et al. (2017) investigated polymeric components from 3D-printed firearms,^156^ while^157,158^ conducted comparative studies using DART-MS and GC-MS, integrating thermal desorption systems with specialized interfaces such as VAPUR. The differentiation between traditional black powder and its substitutes was also achieved through the use of infrared emitters coupled to the DART source.^159^

In summary, the application of DART-MS in forensic science—both for both explosive detection and gunshot residue analysis, represents a highly sensitive, selective, and efficient analytical approach. Its ability to operate under ambient conditions, combined with minimal sample preparation and rapid result acquisition, marks a significant advancement in strengthening criminal investigations. The continuous development of the technique, alongside the integration of novel sampling strategies and instrumental enhancements, reinforces its potential as a vital resource for obtaining accurate and reliable forensic evidence.

## 4 General Discussion and Conclusions

AMS techniques, notably, DESI-MS and DART-MS, have proven to be transformative tools in the field of forensic science. Their ability to carry out fast, direct analyses of complex samples with little to no sample preparation has significantly streamlined many forensic workflows. Over the years, these techniques have been effectively applied in diverse scenarios such as the detection of explosives, gunshot residue, illicit drugs, inks, and even biological fluids. DESI-MS, in particular, stands out for its sensitivity in detecting trace residues on a wide variety of surfaces, while DART-MS offers exceptional efficiency in high-throughput and real-time screening across multiple substrates. Over the past decade, DART-MS has been increasingly employed for wood analysis due to the growing prevalence of illicit timber trade.^160,161^ In this context, Price et al. (2022) demonstrated that DART-MS combined with the ForeST^©^ database provides high reliability and reproducibility in wood species identification, with accurate discrimination at the species, genus, and family levels depending on the chemical complexity of the samples analyzed.^162^

The integration of DESI-MS and DART-MS into criminal investigations has had a meaningful impact on both the scientific and the judicial aspects of forensic work. These technologies allow for the rapid identification of target substances, often directly at the crime scene or under field conditions, contributing to more agile and informed decision-making in time-sensitive cases. Additionally, their nondestructive nature ensures that valuable forensic evidence remains intact for further analysis or legal use. This combination of speed, precision, and preservation not only enhances the reliability of scientific conclusions but also supports more fairer and more robust judicial processes.

However, as with any evolving technology, certain limitations persist. Challenges related to ionization efficiency on specific substrates and the influence of ambient environmental conditions may still require further refinement. Moreover, the absence of standardized analytical protocols and the need for broader validation studies are factors that may restrict the routine use of these techniques in forensic laboratories. Overcoming these obstacles will be crucial to ensure consistency, reproducibility, and legal acceptability of results generated by DESI-MS and DART-MS.

Looking ahead, future research should aim to increase the portability, robustness, and autonomy of AMS instruments, making them even more suitable for field deployment. The integration of artificial intelligence and advanced data processing tools has the potential to enhance compound identification, particularly in complex or unknown mixtures. In parallel, the miniaturization of ion sources and the advancement of imaging capabilities may further expand the role of these techniques in trace evidence analysis, spatial distribution studies, and real-time monitoring applications.

In summary, DESI-MS and DART-MS represent a leap forward in forensic science, offering rapid, reliable, and versatile analytical possibilities. As research continues to push the boundaries of what these technologies can achieve, they are likely to become increasingly central to forensic investigations, strengthening the link between science and justice and reinforcing the role of analytical chemistry in the pursuit of truth. Thus, fostering multidisciplinary collaborations and developing standardized protocols will be key to expanding the routine forensic use of AMS techniques.

In parallel, miniaturized extraction methodologies such as liquid-phase microextraction (LPME) and solid-phase microextraction (SPME) have become increasingly valuable tools in improving the detectability of forensic analytes.^163,164^ These techniques allow for efficient and selective extraction of compounds present at trace levels while significantly reducing the use of organic solvents and the need for extensive sample volumes. Their compatibility with complex matrices and analytical platforms, especially mass spectrometry, makes them particularly well-suited for forensic applications involving biological fluids, environmental samples, and seized materials.^163,165^ When coupled with portable or ambient ionization mass spectrometers, miniaturized extraction methods enhance the analytical performance under field conditions, supporting rapid, sensitive, and eco-friendly workflows. Continued innovation in this area, including the development of reusable sorbents and automation-compatible formats, will be instrumental in expanding their adoption and ensuring more reliable and efficient forensic analyses.
